# A Case Report of Maxillary Second Molar with Two Palatal Root Canals and a Furcal Enamel Pearl

**Published:** 2013-01-20

**Authors:** Shiva Shojaeian, Jamileh Ghoddusi, Sara Hajian

**Affiliations:** 1 Department of Endodontics, Dental School, Shahid Beheshti University of Medical Sciences, Tehran, Iran; 2 Department of Endodontics, Dental Research Center, Dental School, Mashad University of Medical Sciences, Mashad, Iran; 3 Private Practice, Mashad, Iran

**Keywords:** Endodontics, Furcation, Molar tooth, Root canal, Root canal therapies, Tooth root

## Abstract

This case report presents an uncommon case of maxillary molar with two palatal root canals and an enamel pearl in the furcation area. The article discusses root canal complexities of maxillary second molars as well as possibility of coexisting anomalies in the region that makes radiographic interpretation difficult and compromises the success of endodontic treatment.

## 1. Introduction

The main goal of endodontic treatments is prevention of apical periodontitis and/or healing of present periapical lesion. Several factors may influence the treatment success [1]. One of the main causes of failure is residual microorganism in root canal which may reach periapical tissues. So variations of root canal anatomy and morphology are critical for successful endodontic treatment; resulting in thorough root canal cleaning, eradication of microorganisms, and favorable apical seal [2].

Maxillary molars have one of the most complex root canal anatomies. Six different variations of a second maxillary molar have been reported in a retrospective study by Peikoff et al. [3]. These variations include 1) three separate roots and canals (56%), 2) three separate roots and four canals (two canals in mesiobuccal) (22.7%), 3) Three roots and canals with union of mesiobuccal and distobuccal canals to form a common buccal canal (9%), 4) Two separate roots and canals (6.9%), 5) A single root and canal (3.1%) and 6) Four distinct roots and four canals including two palatal canals (1.4%).

Rostein and Libfild have reported a prevalence of 0.4% for second maxillary molar with four roots in a large series of 1200 teeth radiographic assessment [4]. However there are several case reports in literature which report the treatment of second maxillary molar with two palatal canals [5-10].

Second maxillary molars with three buccal roots [11], five roots and canals (two palatal and three buccal) [12], as well as first maxillary molar with three palatal canal [13] have been reported as abnormalities. These variations confirm unexpected root canal morphology in maxillary molars.

Sometimes, complex root canal anatomy may be associated with other anomalies such as enamel pearls or palatogingival groove that may affect both diagnosis and treatment prognosis [14, 15].

An enamel pearl is the ectopic enamel mass which can be seen in the furcation or other area of root surface [16]. Single radicular enamel pearl is more common [17], but two to four may be found in one tooth [18]. Prevalence of enamel pearl in permanent teeth is estimated to be between 1.1-9.7% in different races [19, 20].

This case report presents an uncommon case of maxillary molar with two palatal root canals and an enamel pearl in the furcation.

## 2. Case Report

**Figure 1.**
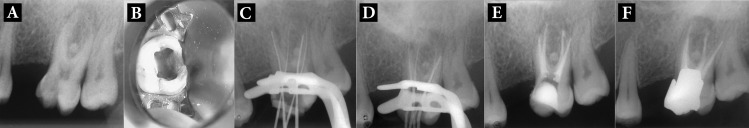
A) Initial radiograph; B) Access cavity; C) Working Length determination radiograph; D) Emphasizing working length by gutta percha cone; E) Final obturation; F) six months postoperative radiograph

A 45-year-old woman came to endodontic department of Mashhad Dental School complaining of sever toothache especially during mastication. The patient medical history was negative. Clinical examination revealed extended mesial decay on the left maxillary second molar, tenderness to percussion and palpation of peri-radicular zone. Vitality tests were negative. Radiographic image (periapical) revealed radiolucency around palatal apex; moreover, there was the opacity in the furcation area which was detected as the enamel pearl (Figure 1A).

The tooth was diagnosed with symptomatic apical periodontitis with necrotic pulp. After 2% Lidocaine with epinephrine 1:100.000 (Persocaine, DarouPakhsh, Tehran, Iran) was administered the tooth was isolated with rubber dam. As usual, in the initial access cavity, three orifices were found, but the palatal one was mesially deviated. So the access cavity was further prepared into a square shape (Figure 1B). Second palatal canal was found after meticulous exploration of the pulp chamber floor with a fine precurved hand K-file. Palatal canals were hardly negotiated with #10K-file (Dentsply, Maillefer, Ballaigues, Switzerland) and RC-Prep (Meta Biomed Co., Seoul, Korea) due to canal calcification. Working length was determined with apex locators ROOT ZX (Morita, Kyoto, Japan) and confirmed radiographically (Figure 1C).

All canals were instrumented with stainless-steel hand k-files accompanied by Flex Master Introfile (VDW, Munich, Germany) rotary instrument using crown-down technique. Root canals were irrigated by sodium hypochlorite 5.25% and dried completely. Calcium hydroxide-distilled water paste was placed as an intra-canal dressing then access cavity was sealed temporarily with Cavit (Ariadent, Tehran, Iran).

After a week, patient was completely asymptomatic. Before root canal obturation, the working length was radiographically confirmed with gutta-percha (Figure 1D). Root canals were obturated with gutta-percha (Meta Biomed Co. Ltd, Cheongju city, Chungbuk, Korea) and AH26 (Dentsply, DeTrey, Konstanz, Germany) sealer using lateral condensation technique (Figure 1E).

Subsequently, patient was referred to restorative department for their final restoration. After six months, patient had no clinical symptoms and complete clinical and radiographic resolution was seen (Figure 1F).

## 3. Discussion

Adequate knowledge and experience can improve the operator’s ability to find additional canals [21, 22] especially in the maxillary molars [23-26]. Furthermore, the age of patient has been considered as the only influencing factor which can significantly affect the number of canals which are found [27].

Christie et al. reported sixteen maxillary molars with two palatal canals during his forty-year experiences in root canal therapy and divided them into three different groups. Type I has two long divergent palatal roots which can be observed radiographically, Type II as two short parallel palatal roots which are mesially and distally divergent in the buccolingual radiographic view and Type III as mesiobuccal, mesiopalatal and distopalatal roots engaged in a web root dentin with radiographic similarities to Type II. Interestingly, the only other anomaly he reported in maxillary molars was double palatal roots due to the presence of an enamel pearl in the furcation of three patients. In one of the cases, it lead to periodontal failure [14].

The present case described maxillary second molar with two palatal root canals with an enamel pearl in furcation region and no periodontal involvement.

Enamel pearls are usually present in furcation area and are more common in second and third maxillary molars [20]. Hertwig’s root sheath which maintains its potential of enamel formation might be responsible for this structure [28]. Enamel pearl in the area would prevent periodontal attachment and predispose the area to pocket formation and periodontal disease [18, 29].

Initial radiographic interpretation of the second maxillary molar root anatomy may be more complicated because of superimposition of roots on each other or adjacent bony structures. Clinicians encounter a dilemma when additional abnormality presents in the area. Thorough knowledge of anatomical variations and frequent anomalies in the region besides multiple radiographs with different angles or cone-beam computed tomography (CBCT) can be helpful [12, 30]. Meticulous exploration of the developmental groove in the pulp chamber floor is suggested in order to locate canals’ orifices; moreover, any dentin projection which could cover existing orifice should be removed carefully [31]. Also, a more mesiodistally extended access may be considered in order to find canal orifices.

## 4. Conclusion

The possibility of two palatal roots and enamel pearls in the furcation should be considered in the second maxillary molars. Though enamel pearls are rare, periodontal evaluation is recommended for early diagnosis.
